# The Use of Hot Water in Hemorrhage Following the Employment of Esmarch’s Bandage

**Published:** 1879-10

**Authors:** 


### THE USE OF HOT WATER IN HEMORRHAGE FOLLOWING THE EMPLOYMENT OF ESMARCH'S BANDAGE.

Dr. Paul Brown, in the August number of the Philadelphia *Medical Times*, reports a case of capillary hemorrhage following the use of Esmarch's bandage for an amputation of the forearm, which bleeding was stopped instantaneously by syringing the parts with hot water (160 Fahr.). His attention was called to the hot-water treatment by Dr. Fordyce Barker's article on the treatment of uterine hemorrhage in that way. The patient had three months previously had his carpus resected by Lister's operation on account of necrosis, and after the operation a very troublesome parenchymatous hemorrhage occurred, which lasted for nearly two hours. It is of interest to know that the patient had a marked hemorrhagic diathesis. That the hot water did no injury to the parts, and did not retard the cure, is demonstrated by the fact that in twelve days from the time of operation, the parts had completely united, and a cicatrix had formed. Dr. Brown thinks that the hemorrhage following the use of Esmarch's bandage probably results from paralysis of the vaso-motor nerves, produced by the pressure of the tense rubber, and that the hot water acts as a powerful stimulant to these nerves, so that they produce a contraction of the arterioles, thus stopping the hemorrhage. Water of a temperature less than 150 Fahr. should never be used. Warm water is worse than useless.

